# Iron deficiency anaemia is still a major killer of pregnant women

**DOI:** 10.12669/pjms.323.9557

**Published:** 2016

**Authors:** Meharun-Nissa Khaskheli, Shahla Baloch, Aneela Sheeba, Sarmad Baloch, Fahad Khan Khaskheli

**Affiliations:** 1 Dr. Meharun-Nissa Khaskheli, MBBS, FCPS. Associate Professor of Obstetrics & Gynaecology, Liaquat University of Medical & Health Sciences Jamshoro, Sindh, Pakistan; 2 Dr. Shahla Baloch, MBBS, DGO, FCPS. Associate Professor of Obstetrics & Gynaecology, Liaquat University of Medical & Health Sciences Jamshoro, Sindh, Pakistan; 3 Dr. Aneela Sheeba, MBBS, DMRD, (FCPS). Assistant Professor of Radiology, Liaquat University of Medical & Health Sciences Jamshoro, Sindh, Pakistan; 4 Dr. Sarmad Baloch, MBBS. House Officer, Medical Department, Liaquat University of Medical & Health Sciences Jamshoro, Sindh, Pakistan; 5 Dr. Fahad Khan Khaskheli, MBBS. House Officer, Medical Department, Liaquat University of Medical & Health Sciences Jamshoro, Sindh, Pakistan

**Keywords:** Severe anaemia, High maternal morbidity, Mortality

## Abstract

**Objective:**

To observe the effects of iron deficiency anaemia on the health and life of pregnant women.

**Methods:**

This cross sectional study was conducted at the Department of Obstetrics and Gynaecology Unit IV, Liaquat University of Medical and Health Sciences Jamshoro from 1st June 2015 to 30^th^ November 2015, for the period of 6 months. During this study period all the pregnant women from 13-40 weeks of pregnancy with iron deficiency anaemia having haemoglobin level less than 9 gram% were included, while the pregnant women with other medical disorders were excluded from the study. The data was collected and analyzed on SPSS version 21.

**Result:**

Out of the 305 pregnant registered women with iron deficiency anaemia most women were young 170(55.73%) between 20-30 years, belonged to low socioeconomic group 254(83.27%), they were multiparous 104(34.09%), having very low haemoglobin level between 1-3 gram % in 54(17.70%) women and between 4-6gram% in162 (53.11%) women. These women were prone to high complications such as ante partum haemorrhage 49(16.06%), renal failure 48(15.73%), disseminated intravascular coagulation 54(17.70%) and 16(5.24%) women died.

**Conclusion:**

Iron deficiency anaemia is common in pregnant women with higher rates of complications.

## INTRODUCTION

Anaemia is a global public health problem affecting developed and adversely affecting developing countries. About 1.62 billion peoples are affected worldwide, currently pregnant women are the most vulnerable population corresponding to 24.8%.^1^,^2^ The highest prevalence of anemia exists in the developing world, where its causes are multi-factorial.^3^

The prevalence’s of anaemia varies according to the socio-economic status, dietary deficiencies, Cultural taboos,infections, multiple pregnancies, low contraceptive prevalence, and with all these haemoglobinopathies are additional factor.^4^ Common type of anaemia is iron deficiency, this is an nutritional deficiency disorder and the pregnant women are highly vulnerable population particularly with its frequent risks even with first pregnancy.^5^ Anaemic pregnant women are prone to severe morbidity and mortality in developing countries.^6^ Consequences with milder form of anaemia are “silent”, without symptoms. In its severe form, anaemia is associated with symptoms like fatigue, weakness, dizziness and drowsiness. It may further include loss of normal colour in the skin (in fair skinned people) and also in the lips, tongue and nails. In Asia, anaemia (irrespective of the severity) is the second leading cause of maternal death accounting 12.8% independent of deaths due to postpartum haemorrhage.^4^ Literature search further adds that about 20% of maternal deaths are caused by anaemia and with this anaemia is additional risk factor in contribution of 50% of all maternal deaths.^7^,^8^ There are three main reasons for death due to anaemia First, anaemia results from excessive blood loss during or after delivery resulting in low haematological reserves. Second, with severe anaemia resistance is decreased and susceptibility to infection is increased; and third, haemoglobin (Hb) level of less than 4 g/dl is associated with high risk of cardiac failure and death particularly during delivery or soon after.^9^

The rationale of the study was to determine the magnitude of the anaemia related morbidities which are avoidable and to enforce new strategies for the prevention of the problem as well as awareness regarding the life threatening complications. There is strong need to pay more attention than currently it is receiving, such types of studies will play a vital role in improving the awareness and proper management at earliest so as to decrease the complications resulting from this preventable health disorder.

## METHODS

During this study period the subjected study population included were 305 pregnant women referred from different hospitals rural and urban areas with iron deficiency anaemia and associated complications or acute symptoms at different gestational ages, their haemoglobin level varied between 1-9 gram % and their gestation period was between 13-40 weeks. The sample size was estimated in empirical way with incidence, 27.2%,^10^ confident interval, 95%, and applying formula: N= (Z) ^2^(pq)/e^2^=305. Sampling technique was non probability convenience sampling. These women were registered after taking informed written consent and taking approval from institutional ethic research committee. These women were evaluated in detail regarding their age, parity, socioeconomic status, gestational period, symptomatology, thorough general, physical examination was done, colour of skin, nails, conjuctiva was seen, for confirmation of the type of anaemia, relevant investigations were carried out such as complete blood picture with absolute values, serum ferritin level, haemoglobin electrophoresis considering their cost effectiveness. Those pregnant women with other metabolic or chronic disorders were excluded from the study. All these women were managed according to the institutional management protocol. The case records of all these women were recorded on the predesigned proforma. The data was collected and analyzed on statistical package of social sciences (SPSS) Version 21(IBM SPSS 21, Inc., USA, 2012). The result is presented in forms of simple frequency and percentage for the categorical data such as age, parity, gestational period mean±standard deviation is given, chi square test was applied for qualitative type of analysis, P-value >0.05 was considered significant.

## RESULTS

Out of 305 pregnant anaemic women, 254(83.27%) women belonged to low socio economic group, while middle class women were 50 (16.39%). Majority of these women were between 21-30 years age group 170(55.73%), while 75(24.59%) women were above 31 years and 60 (19.67%) women were younger having age below 20 years, mean age±standard deviation was 26.50±6.357. Most commonly these women were grand multiparous 105(34.42%), primi gravid women were 52 (17.04%), (Table-I). Common mode of presentations were generalized oedema 87 (28.52%), dyspnoea 72(23.60%), weakness 46(15.08%). These women presented most frequently at 31-40 weeks gestational period comprising 158 (51.80%). The haemoglobin level of these women varied between 4-6 gram% in 162 (53.11%) women, 7-9 gram % in 89(29.18%) women, while 54(17.70%) women were having haemoglobin level between 1-3 gram%, majority of these women were having serum ferritin level between 12-70ng/ml. Table-II. Most frequent complication seen were ante partum haemorrhage in 74(60.16%) women and majority of these women were having haemoglobin level between 7-9gram%, while post partum haemorrhage was observed in 42(66.66%) women and these women were having heamoglobin level between 4-6 gram%, and in these women disseminated intravascular coagulation rate was also most frequently observed 42(76.36%). The mortality rate was 16(5.24%), out of these 10 (62.5%) women were having haemoglobin level between 1-3 gram% and in 6(37.5%) women haemoglobin level varied between 4-6 gram%. (Table-III).

**Table-I:** Sociodemographic characteristics N=305.

*S/N*	*Socio demographic characteristics*	*No. of cases*	*Percentage*	*P-value*
1.	*Socio economic status*			
	a. Low	254	83.27	0.277
	b. Middle	50	16.39	0.302
	c. High	1	0.327	0.028
2.	*Age*			
	a. <20 years	60	19.67	0.054
	b. 21-30 years	170	55.73	0.053
	c. 31 years and above	75	24.59	0.024
3	*Parity*			
	a. Primigravid	52	17.04	0.584
	b. para 1-3	94	30.81	0.556
	c. Para 4-5	105	34.42	0.529
	d. Para>6	55	18.03	

**Table-II:** Clinical presentation and gestational period N=305.

*Clinical presentation*	*No of cases*	*Percentage*	*P-Value*
a. Dyspnoea	72	23.60	0.000
b. Weakness	46	15.08	0.000
c. Unable to Perform Routine Work	8	2.62	0.000
d. Generalized oedema	87	28.52	0.000
*Gestational period*
a. 13-20 weeks	22	7.18	0.000
b. 21-30 weeks	125	40.98	0.000
c. 31-40 weeks	158	51.80	0.000

**Table-III:** Haemoglobin level versus morbidities and mortality N=305.

*Haemoglobin level*	*Morbidities*				*Mortality*	*P-Value*

	*Antepartum haemorrhage*	*Post partum haemorrhage*	*Renal failure*	*Disseminated intravascular coagulation*		
a.1-3 gram% 54(17.70%)	10(8.13%)	7(11.11%)	15(31.25%)	12(21.81%)	10(62.5%)	0.000
b.4-6 gram% 162(53.11%)	39(31.70%)	42(66.66%)	33(68.75%)	42(76.36%)	6(37.5%)	0.000
c.7-9 gram % 89(29.18%)	74(60.16%)	14(22.22%)	0	1(1.81%)	0	0.000

Total: 305	123(40.32%)	63(20.65%)	48(15.73%)	55(18.03%)	16(5.24%)	

## DISCUSSION

Even after implementation of different programs for controlling iron deficiency anaemia, the magnitude of the problem is still high. Current literature is focusing it less as it is chronic problem and there is no improvement with folic acid and iron supplementation. Iron deficiency anaemia is more prevalent especially in low socio economic group because of improper nutrition, living in unhyiegenic condition, high infection rate, lac of health care facilities and the proper utilization of them, women empowerment, lac of education, early marriage, no birth spacing, no knowledge of ante natal care, these all are important contributing factors. In this study the prevalence of iron deficiency anaemia was high in low socioeconomic group 254 (83.27%) comparing with the result of other studies,^11^,^12^ report is same regarding the prevalence of anaemia and its associated contributing factors. Iron deficiency anaemia was more frequently observed in women between 21-30 years of age 170(55.73%), the main reason could be nutritional deficiency, already depleted iron store before pregnancy, high infection rate same is reported by Viveki et al and Judith AN studies.^13^-^15^ In this study higher prevalence of iron deficiency anaemia was seen in Para 4-5 women 105(34.42%). The number of pregnancies (gravidity) is an important variable significantly associated with anemia. The risk of anaemia increases as the number of pregnancies increases from 3–5 pregnancies, but it is even common in the women who had less than three pregnancies. This finding is consistent with other studies conducted in Saudi Arabia and India, in which they found that increased number of pregnancies and deliveries is positively associated with the high risk of developing anemia.^6^,^16^ This could be due to the loss of iron and other nutrients during increased and repeated pregnancies and also the possibility of sharing of resources with the fetus. These women presented commonly with generalized oedema 87(28.52%), weakness 42(15.08%), dyspnoea 72(23.60%), unable to perform routine work 8(2.62%). In milder form anaemic pregnant women will remain symptoms free but as its severity increases women will have symptoms.^3^ At the time of presentation most of the reported anaemic mothers 158(51.80%) were in the third trimester of pregnancy, since the iron demand reaches 6.6 mg/day in this period and during this gestational period frequency of antepartum haemorrhage was higher. There is an increase in plasma volume and red cell mass, especially in third trimester. But the disproportionate increase in plasma volume causes haemodilution and it lowers the haemoglobin level. The risk of developing anemia increases with the age of pregnancy (trimester).

The risk of developing anemia was higher in third and second trimester when compared with those in the first trimester. This finding is consistent with a study done in Saudi Arabia, which found that the prevalence of anemia is higher in the third trimester in comparison with first trimester,^6^ and another study conducted in India, which also indicated that the prevalence of anemia was higher in pregnant women in the third and second trimesters.^16^ Additionally, studies conducted in Malaysia, Vietnam, and Nepal found that increased gestational age is significantly associated with the risk of developing anemia.^17^ This could be due to the fact that when the gestational age increases the mother becomes weak and the iron in the blood is shared with the fetus in the womb therefore decreasing the iron binding capacity of the mother’s blood. Mortality rate was higher in the women with very low haemoglobin level and with associated co-morbidities such as postpartum haemorrhage, renal failure, and disseminated intravascular coagulation. same is reported by other studies.^7^,^18^-^21^

**Fig.1:**
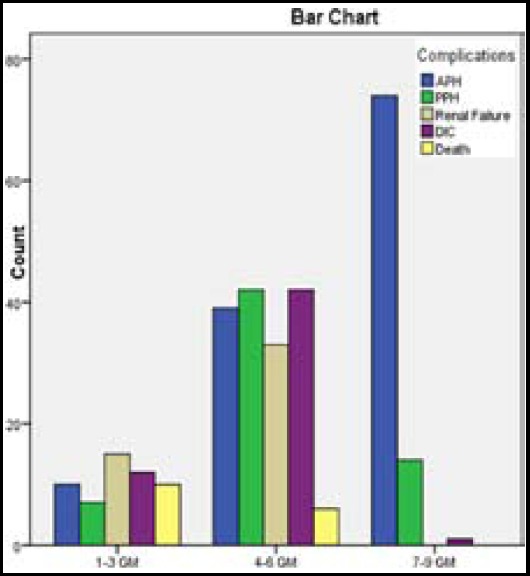
Haemoglobin Level (Iron deficiency anaemia).

## CONCLUSION

Iron deficiency anaemia is common during pregnancy, high prevalence was seen in late pregnancy, and severity of anaemia is associated with high rate of morbidity and mortality.

## References

[1] Hannif J, Das A, Onn LT, Sun SW, Nordin NM (2007). Anemia in pregnancy in Malaysia. cross-sectional survey. Asia Pacific J Clin Nutr.

[2] Alem M, Enawgaw B, Gelaw A, Kenaw T, Seid M, Olkeba Y (2013). Prevalence of Anemia and Associated Risk Factors among Pregnant Women Attending Antenatal Care in Azezo Health Center Gondar Town. Northwest Ethiopia J Interdiscipl Histopathol.

[3] Kawaljit Kaur BD (2014). Anaemia ‘a silent killer’ among women in India. Euro J Zool Res.

[4] Van den Broek NR, White SA, Neilson JP (1998). The relationship between asymptomatic human immunodeficiency virus infection and the prevalence and severity of anemia in pregnant Malawian women. Am J Trop Med Hyg.

[5] Ahmad N, Kalakoti P, Bano R, Syed MMA (2010). The prevalence of anaemia and associated factors in pregnant women in a rural Indian community. Aust Med J.

[6] Elzahrani SS (2012). Prevalence of iron deficiency anemia among pregnant women attending antenatal clinics at Al-Hada Hospital. Canadian J Med.

[7] Sanghvi TG (2010). Harvey. Maternal iron-folic acid supplementation program:Evidence of impact and implementation. Food Nutr Bull.

[8] Galloway R, Dusch E, Elderet L (2002). Women’s perceptions of iron deficiency and anemia prevention and control in eight developing countries. Sci Dir Soc Sci Med.

[9] Buseri FI, Uko EK, Jeremiah ZA, Usanga EA (2008). Prevalence and Risk Factors of Anaemia Among Pregnant women in Nigeria. Open Hematol J.

[10] Naz H, Begum B (2013). Prevalence and associated risk factors of anaemia in pregnant women in a teaching hospital, Korangi Industrial Area. Pak J Surg.

[11] Puspa O, Vinod DK, Prakash LG, Ashok PK (2012). A study of prevalence of anemia and sociodemographic factors associated with anemia among pregnant women in Aurangabad city. India.

[12] Ivan EA, Mangaiarkkarasi A (2013). Evaluation of anaemia in booked antenatal mothers during the last trimester. J Clin Diagn Res.

[13] Viveki RG, Halappanavar AB, Viveki PR, Halki SB, Maled VS, Deshpande Al (2012). Prevalence of Anaemia and Its Epidemiological Determinants in Pregnant Women. J Med Sci.

[14] Angelittanoronha J, Al-Khasawneh E, Seshan V, Ramasubramaniam S, Raman S (2012). Anemia in pregnancy- Consequences and challenges:A Review of literature. J South Asian Federation Obstet Gynaecol.

[15] Ghazala N, Saima N, Shafqut A, Shaheen A, Malik SA, Qari IH (2011). Anaemia:the neglected female health problem in developing countries. J Ayub Med Coll Abbottabad.

[16] Vivek RG, Halappanavar AB, Vivek PR, Halki SB, Maled VS, Deshpande PS (2012). Prevalence of Anemia and its epidemiological. Determinants in Pregnant Women.

[17] Makhoul Z, Taren D, Duncan B (2012). Risk factors associated with anemia, iron deficiency and iron deficiency anemia in rural Nepali pregnant women. Southeast Asian J Trop Med Public Health.

[18] Ghimire RH, Ghimire S (2013). Maternal and fetal outcome following severe anaemia in pregnancy:Results from Nobel Medical College Teaching Hospital, Biratnagar, Nepal. J Nobel Med Coll.

[19] Wandabwa J, Dovle P, Todd J, Ononge S, Kiondo P (2008). Risk factors for severe postpartum hemorrhage in Mulago Hospital, Kampala, Uganda. East Afr Med J.

[20] Kaima A (2015). Frass. Postpartum hemorrhage is related to the hemoglobin levels at labor:Observational study. Alenxdria J Med.

[21] Kalaivani K (2009). Prevalence and consequences of anaemia in pregnancy. Indian J Med Res.

